# Transcription Factor *CsWIN1* Regulates Pericarp Wax Biosynthesis in Cucumber Grafted on Pumpkin

**DOI:** 10.3389/fpls.2019.01564

**Published:** 2019-11-29

**Authors:** Jian Zhang, Jingjing Yang, Yang Yang, Jiang Luo, Xuyang Zheng, Changlong Wen, Yong Xu

**Affiliations:** ^1^Beijing Vegetable Research Center (BVRC), Beijing Academy of Agricultural and Forestry Sciences, National Engineering Research Center for Vegetables, Beijing, China; ^2^Beijing Key Laboratory of Vegetable Germplasms Improvement, Beijing, China

**Keywords:** pericarp wax, transcriptome, methylation, *CsWIN1*, grafted cucumber

## Abstract

Pericarp wax of cucumber is an important economic trait, determining sales and marketing. Grafting of cucumber onto pumpkin rootstock (*Cucurbita moschata*) is an effective way to produce glossy cucumber fruits. However, the molecular regulation mechanism of this phenomenon remains largely unknown. In the present study, transcriptome analyses, genome-wide DNA methylation sequencing, and wax metabolite analysis were performed on the pericarp of self-rooted versus grafted cucumber. We identified the AP2/ERF-type transcription factor *CsWIN1* as methylated and significantly upregulated in grafted cucumber compared to self-rooted cucumber. The increased expression of *CsWIN1* was also positively correlated with several key wax biosynthesis genes, including *CsCER1*, *CsCER1-1*, *CsCER4*, *CsKCS1*, and the wax transporter gene *CsABC*. The transcriptome expression level of these genes was validated through qRT-PCR profiles. Furthermore, wax metabolite analysis showed that more wax ester (C20 fatty acid composition), but fewer alkanes (C29 and C31) were deposited in grafted cucumber pericarp. The higher expression of *CsWIN1* and wax biosynthesis genes was reflected in the glossier appearance of grafted pericarp, possibly the result of higher wax ester content and higher integration of small trichomes in the pericarp. This study demonstrates that grafting can affect the content and composition of pericarp wax in cucumber grafted on pumpkin, and a unique regulation model of *CsWIN1* for wax biosynthesis may exist in cucumber.

## Introduction

Cucumber is one of the major vegetables cultivated worldwide (Huang et al., 2009). China is the largest producer and consumer of cucumber with about 77.4% of the total cucumber yield in the world (Year 2017, www.fao.org/faostat/en/).

Glossy cucumber is a dominant player in the Chinese market. The pericarp appearance of cucumber is an important economical factor, appealing to customers and plays an important role in sales and marketing of the fruit worldwide. The pericarp wax is mainly composed of wax and cutin derived from the cuticle and is a critical trait affecting the appearance and quality of cucumber (Wang et al., 2015b; Li et al., 2019). The waxy cucumber has a natural white powder-like substance on the pericarp surface, which is less desirable than cucumber varieties with a glossy pericarp.

Tian et al., (2015) identified five QTL (*WP1.1*, *WP3.1*, *WP5.1*, *WP6.1* and *WP6.2*) promoting pericarp wax accumulation and two QTL (*WP5.1* and *WP6.2*) with moderate effect. Other researchers revealed that the pericarp wax in cucumber is affected by both potential biosynthesis genes (*CsCER1*, *CsWAX2*) and environmental factors, such as light intensity, temperature, moisture, and grafting (Liu et al., 2014; Wang et al., 2015b; Wang et al., 2015c). However, there has been little research focusing on the genetic mapping of pericarp wax biosynthesis genes, which are critical to the discovery of the pericarp wax biosynthesis pathway in cucumber. Grafting onto pumpkin rootstock (*Cucurbita moschata*) proved to be an effective way to brighten the pericarp of cucumber (Samuels et al., 1993; Buschhaus et al., 2015; Shen et al., 2015). Still, the molecular mechanisms in regulating pericarp wax biosynthesis in cucumber grafted on pumpkin is unknown. There is a need to elucidate the biosynthesis pathway of pericarp wax in cucumber and identify the gene loci that confer glossy appearance in cucumber.

The cuticle layer covers the epidermal cells of leaves and other plant parts. It protects the plant from various environmental stresses, including fungal pathogens, UV radiation, water loss and non-stomatal transpiration, and dust deposits (Kunst and Samuels, 2009; Buschhaus et al., 2015; Blanc et al., 2018).The chemical components of cuticular wax has been well studied in *Arabidopsis* and other crops. Several functional genes involved in wax biosynthesis (*CER1*, *WAX2*, and *FAC4*) and transportation (ABC/LTP transporters) were identified (Broun et al., 2004; Suh et al., 2005; Bernard et al., 2012; Park et al., 2016). Cuticular wax was shown to be biosynthesized from C16 and C18-CoAsin plastids and then elongated into a long chain fatty acids (VLCFAs, chain length >20 carbons) in the endoplasmic reticulum membrane (Suh et al., 2005; Kunst and Samuels, 2009). Cuticular wax covers the outer surface of the cutin layer and presents in the form of crystals, a complex mixture that contains various primary and secondary alcohols, alkenes, ketones, and esters derived from VLCFAs through alkane- and alcohol-forming pathways (Lee et al., 2009; Gan et al., 2016). However, the chemical composition and content of cuticular wax are different among species and even tissues (Kunst and Samuels, 2009). Moreover, several studies have found that cuticular wax biosynthesis is affected by environmental factors, such as water deficit, lower temperature, and high levels of UV light (Kunst and Samuels, 2009; Lee and Mi, 2015). AP2-ERF (APETALA2/ethylene) transcription factors (TFs) were involved in the regulation of ET-response genes and had proved to possess a variety of functions in plants (Gutterson and Reuber, 2004; Feng et al., 2005; Li et al., 2011; Huang et al., 2016). Among these, *WIN1* was first identified in a mutant in *Arabidopsis thaliana*; the overexpression of *WIN1* caused a glossy phenotype, indicating that *WIN1* is a regulator of wax biosynthesis, and it was reported to activate the expression of wax biosynthesis genes such as *CER1*, *CER2*, and *KCS1*(Broun et al., 2004). The other AP2/ERF gene *DEWAX* was found to negatively regulate cuticular wax biosynthesis in *A. thaliana* (Go et al., 2014). Furthermore, a positive regulator of wax biosynthesis “*WRI4*”, a member of the ERF family was identified in *Arabidopsis* stems (Park et al., 2016). There is a need to determine the role of each of the AP2/ERF transcription factors in regulating wax biosynthesis in cucumber.

Glossy cucumber is a dominant player in the Chinese market. Grafting is used for defending cucumber plants against soil-borne diseases. At the same time, it is an important method for production of glossy cucumbers. In this study, the cucumber variety “Jingyan 118” was grafted onto the pumpkin rootstock variety “Jingxinzhen 6,” an elite line used in grafting to brighten the scion cucumber pericarp. We conducted associated transcriptome and genome-wide methylation analyses in conjunction with changes in the cucumber pericarp wax in response to grafting. We determined that the *CsWIN1* gene of cucumber (homologous to *WIN1* in *Arabidopsis*) is methylated and upregulated and at the same time transcriptionally regulates several wax biosynthesis genes, including *CsCER1* and *CsCER4* in the pericarp of grafted cucumber.

## Materials and Methods

### Plant Materials and Grafting Experiment

The experiment was conducted in the Beijing Vegetable Research Center (BVRC) from 26 March to 10 July, 2017. Seeds of the cucumber variety “Jingyan 118” with high pericarp wax and a pumpkin rootstock “Jingxinzhen 6” were sown in a standard potting mix (peat: sand: pumice, 1:1:1, V/V/V). The cuttage grafting system was applied when the scions were growing at the one true leaf and the cotyledon of the rootstock was in the expansion stage. To enhance the survival rate, grafted seedlings were kept in the shade (24–28°C, 80–90% RH) for 3 days. Two weeks later, self-rooted and grafted seedlings were transplanted into soil in a greenhouse. Fertilization and cultivation management methods were as commonly recommended in cucumber production. The pericarp of self-rooted and grafted cucumber at commodity maturity were extracted for the following experiments below.

### Pericarp Wax Observation and Chemical Component Analysis

Using a sharp thin blade, a 1 cm^2^ pericarp was carefully cut off from cucumber fruits at marketable mature stage. Images of the cuticular wax crystals were visualized at 200× magnification using a scanning electron microscope (Semagn et al., 2014) (S4700, Hitachi, Japan). Cellular morphology under the microscope was also observed using cryosection techniques. Long alkanes analysis of wax by gas chromatography was performed, following a method described by Park (Park et al., 2016). Wax esters containing saturated and unsaturated fatty acids from five biological replicates were detected by using specific multiple-reaction monitoring (MRM) scanning (Lam et al., 2013).

### RNA Isolation and Library Construction

Total RNA of the pericarp from self-rooted, grafted, and failed grafted cucumber (cucumber scion which developed roots connected with the stock pumpkin and/or raised new roots into the soil) with three biological replicates were extracted using a DNeasy Kit and miRNeasy Kit, respectively (QIAGEN, USA). The concentration and quality of DNA and RNA were evaluated by a NanoDrop 2000C Spectrophotometer and an Agilent 2100 Bioanalyzer. Pyro-sequencing assays were designed and performed by BIOMARKER Company with both programs and assay result data supplied. mRNA was isolated by Oligo-dT magnetic beads from RNA, then the cDNA was synthesized using a QiaQuick PCR Extraction Kit (QIAGEN, USA). The cDNA library was constructed and sequenced by Illumina Hiseq 2500.

### Differential Expressed Genes Analysis

Raw sequencing reads containing adaptors and low-quality (Q30 < 85%) were filtered. Then the remained reads were aligned to the genome of cucumber (9930 Version2) with TopHat2 (Kim et al., 2013), which mismatch was set as 2 and other parameters as the default value. FPKM (Fragments per Kilobase of transcript per Million fragments mapped) was used to detect the transcript abundance of each gene and estimate the expression values in all samples (Trapnell et al., 2010). Differentially expressed genes (DEGs) were identified according to the following criteria of |log2(fold change)| > 1 and false discovery rate (FDR) < 0.01 by DESeq2 (Love et al., 2014).

### Genomic Methylation Analysis of Pericarp in Grafted Cucumber

In this study, the genome-wide methylation sequencing method was used according to the protocol described by Wang et al. (2015a), which was proved as a simple and scalable method. Total DNA of pericarp from self-rooted, grafted, and failed grafted cucumber with three biological replicates was extracted using a DNeasy Kit. According to the AFSM methylation technology, two restriction enzyme pairs of EcoRI-MspI and EcoRI-HpaII were used in the treatment of cucumber pericarp DNA samples in this study (Xia et al., 2014). The isoschizomers EcoRI was used as a frequent cutter while MspI and HpaII were used as rare cutters, and the methylation-susceptible sequences 59-CCGG and their methylation statuses were assessed by the AFSM method (Xia et al., 2014). The results of AFSM methylation were based on comparisons of the EcoRI-MspI and EcoRI-HpaII assembled sequences at the 59-CCGG sites using custom Perl scripts (http://afsmseq.sourceforge.net/) for each DNA sample analyzed by the China Golden Marker (Beijing) biotechnology company.

### Expression Validation of Differentially Expressed and Methylated Genes

Quantitative real-time RT-PCR (qRT-PCR) technology was employed to verify the expression level of some candidate genes from RNA-seq results. The first-strand cDNA was amplified using a SYBR Green PCR Master Mix Kit (TaKaRa, Japan). Primer sequences for RT-PCR were designed on the “Quant Prime” website (http://quantprime.mpimp-golm.mpg.de/) (**Table S1**). qRT-PCR was conducted on an Applied Biosystems 7500 RT-PCR system (Thermo Fisher, USA) according to the manufacturer’s instruction. The relative transcript levels of candidate genes and *TUA* (internal control) were calculated using the 2^−ΔΔCt^ method. Three biological and technical replicates were performed; the differential gene expression level was determined by t-test (*p* < 0.01).

### Transcriptional Regulation Validation of *CsWIN1* in Pericarp Wax Biosynthesis

To investigate the transcriptional regulation of *CsWIN1*, cDNA of *CsWIN1* was amplified using specific primers (**Table S1**) and constructed into the vector pGADT7-Rec. The promoter sequences of four targeted genes (*CsCER1*, *CsCER1-1*, *CsCER4*, and *CsKCS1*) were amplified from genomic DNA and transformed into the pAbAi vector. Then the linearized library vectors pGADT7-Rec and pAbAi-HYT were co-transformed into the yeast strain Y1HGold. The transformed yeast cell with an empty pGADT7 vector was set as a negative control. The cultivation and transformation of yeast were carried out as described in the manufacturer’s protocol. All primers used in this study are list in **Table S1**.

## Results

### Observation of Pericarp Wax in Grafted Cucumber

**Figure 1:**
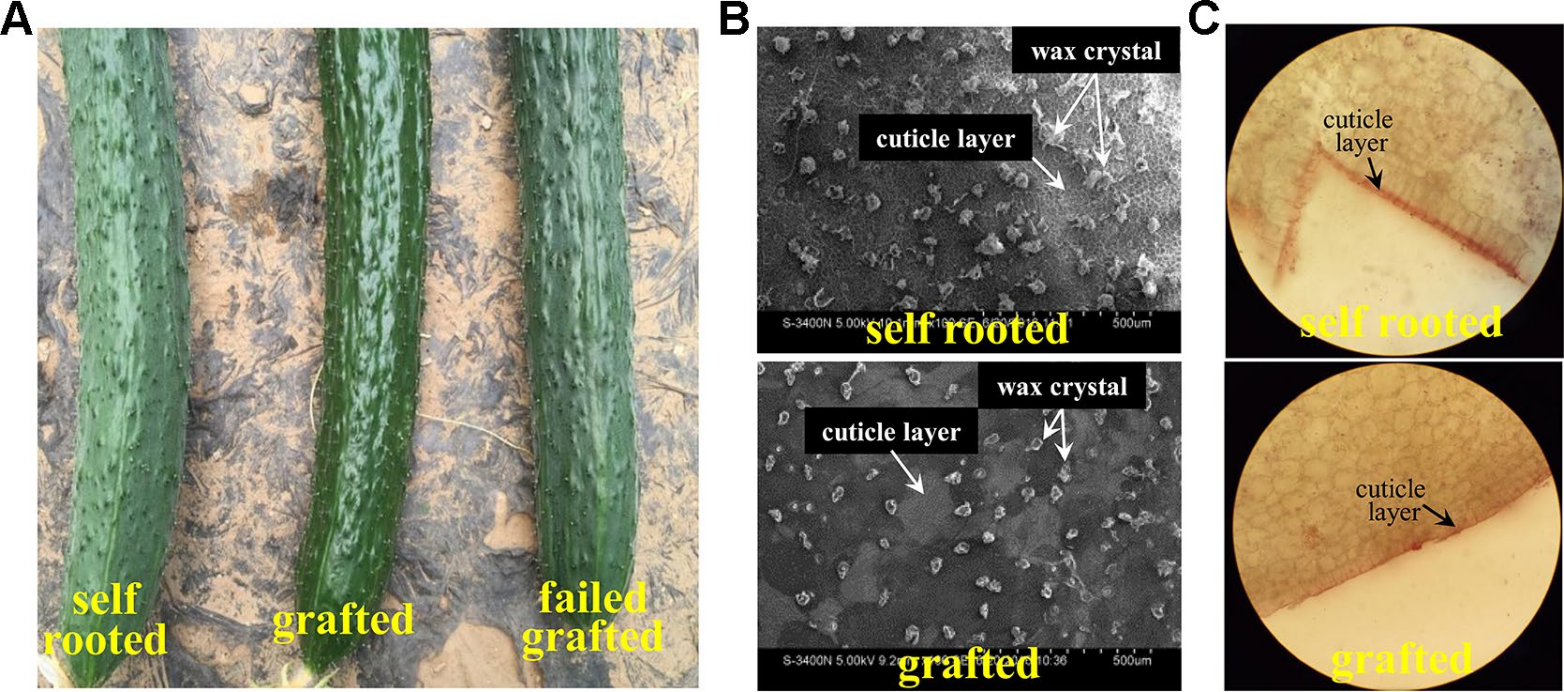
Observation of pericarp appearance in grafted cucumber and in self-rooted cucumber. **Figure 1A** shows the pericarp of self-rooted, grafted, and failed grafted cucumber. The self-rooted and failed grafted pericarp samples were waxy while the grafted cucumber pericarp was glossy. **Figure 1B** displays the SEM observation of self-rooted and grafted cucumber pericarp. The small trichomes of self-rooted pericarp were broken, while those in the grafted pericarp were integrated. **Figure 1C** presents the frozen pathological examination. The cutin in self-rooted pericarp was found to be much thicker than in grafted cucumber.

The grafted cucumber exhibited a glossy pericarp with a light green color, in contrast to the self-rooted and failed grafted cucumber (**Figure 1A**). SEM (scanning electron microscope) observation revealed that the content and form of the wax was significantly different in grafted cucumber versus self-rooted cucumber. Fewer wax crystals were detected on the fruit surface, and the form of the wax was smaller and unbroken in grafted cucumber (**Figure 1B**). Wax crystals were more abundant in self-rooted and failed grafted cucumber and the wax exhibited a broken form, expressed in rods and tubes (**Figure 1B**). Cryosectioning that was conducted in both self-rooted and grafted cucumber showed that a thicker cuticle (15mm × 15mm) was clearly observed in self-rooted cucumber (**Figure 1C**).

### Components Analysis of Pericarp Wax in Grafted Cucumber

**Figure 2:**
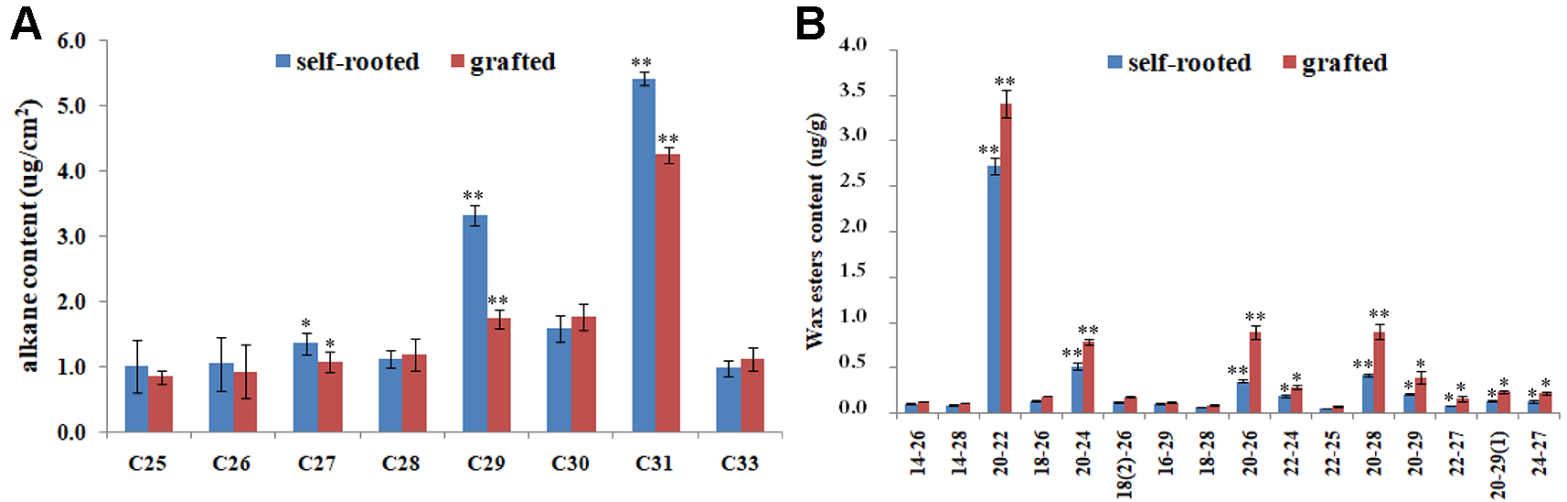
Comparison of the composition of pericarp wax between self-rooted and grafted cucumber, analyzed by GC-MS. The major alkanes in **Figure 2A** were designated by carbon chain length in *x* axis and the amount of alkanes were expressed as μg/cm^2^ pericarp area in *y* axis. The numbers in **Figure 2B** showed the carbon chain length of fatty acid and fatty alcohol in *x* axis, the corresponding wax esters amount were expressed as μg/g in *y* axis. Numbers in brackets indicated the amount of double bond. Error bars indicate standard errors. * and ** indicated significant differences of *t* test at *p* < 0.05 and *p* < 0.01, respectively.

The significantly reduced formation of wax powder in grafted cucumber prompted us to investigate the changes in chemical composition of cuticular wax using GC-mass spectrometry at the commercial maturity stage. We detected a significant decrease in the content of alkanes with length of 29 and 31 carbons (which are important components of wax) in grafted cucumber pericarp compared with the self-rooted and failed grafted cucumber (**Figure 2A**). The levels of these two alkanes components were decreased in the grafted versus self-rooted cucumber by approximately 50 and 30%, respectively. Grafted cucumber contained increased amounts of wax esters as compared to self-rooted cucumber (*p* < 0.01), especially these wax esters derived from C20 fatty acids, such wax ester C20:C22, wax ester C20:C24, wax ester C20:C26, wax ester C20:C28, and wax ester C20:29 (**Figure 2B**). Total wax ester levels were increased by approximately 15% in grafted cucumber compared to self-rooted cucumber. No noticeable changes in other alkanes and wax esters were detected in the pericarp of grafted cucumber versus self-rooted cucumber.

### Transcriptome Analysis of Pericarp Wax Biosynthesis After Grafting

Nine cucumber cDNA libraries from self-rooted, grafted, and failed grafted cucumber were sequenced on the Illumina HiSeq2500 platform; and 33.87 Gb clean reads were generated in total. Each library generated about 4.84 Gb clean reads; all Q30 reached more than 94.69% (**Table S2**). All of the clean sequencing data used in the present study are deposited in the NCBI Sequence Read Archive database (SRA accession: SRR10113591, SRR10113592, SRR10113593, SRR10113594, SRR 10113569, and SRR10113570). The sequencing reads from nine libraries showed a significant positive correlation (0.98) among the three replications, indicating the reliable phenotype variations and high-quality sequencing data (**Figure S1**). All clean reads were aligned to Chinese long cucumber reference genome (9930 V2) and the mapping rate varied from 87.91 to 89.51% (**Table S2**). Then, all mapped reads were aligned with the databases of Swiss-Prot, KEGG, and GO using BLAST. Four hundred and twenty-four new genes were identified, 347 of which were annotated depended on the test for functional annotation.

**Figure 3:**
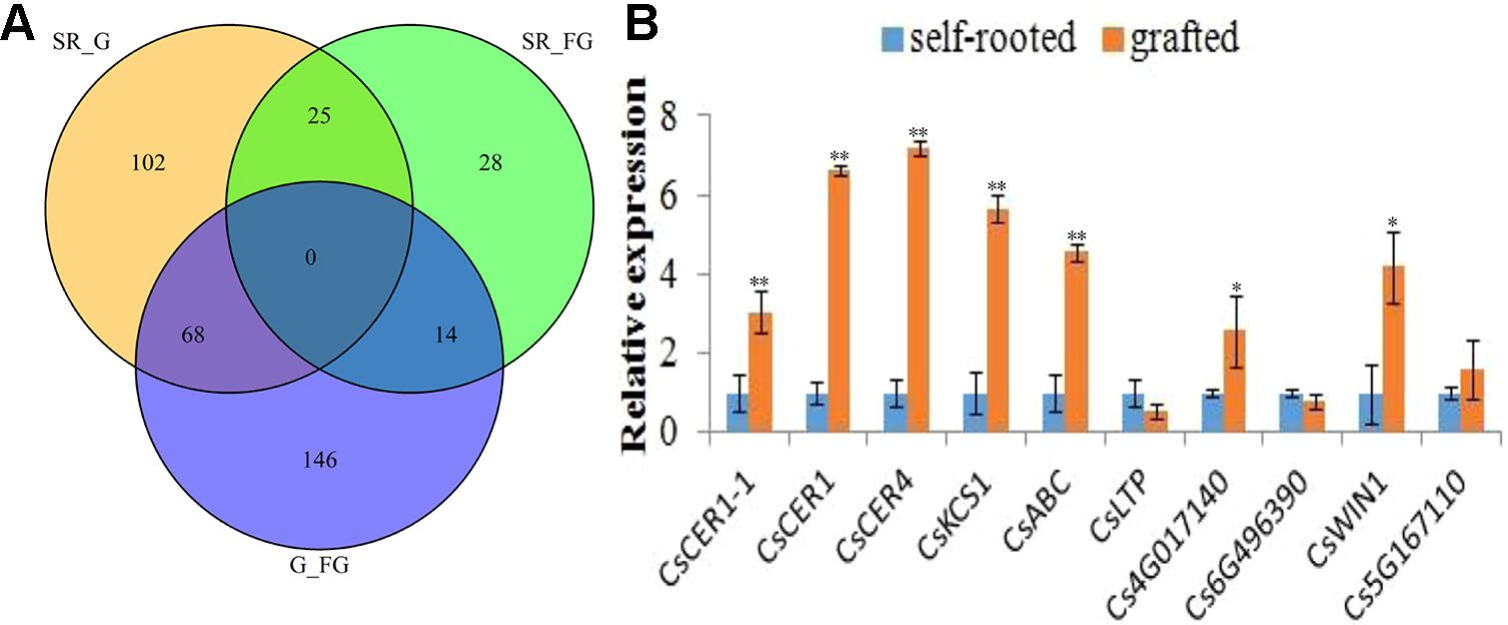
Transcriptome dataset and expression validation of differentially expressed genes affected by grafting at commercial mature stage. **Figure 3A** presents a Venn diagram of differentially expressed genes (DEGs) obtained from the self-rooted, grafted, and failed grafted cucumber pericarp. **Figure 3B** shows the qRT-PCR validation (three technical replicates per biological replicate) of the 10 differentially expressed genes involved pericarp wax biosynthesis. Error bars indicate the standard deviations. * and ** indicated significant differences of *t* test at *p* < 0.05 and *p* < 0.01, respectively.

Differentially expressed genes (DEGs) were identified by calculating the FPKM values among self-rooted, grafted, and failed grafted cucumber with the following criteria (*p* < 0.05, FDR < 0.01). Finally, a total of 384 DEGs were identified in this study (**Figure 3A**). Compared with self-rooted cucumber, 111 genes were upregulated while 82 genes were downregulated in grafted cucumber. In contrast to failed grafted cucumber, 118 genes were upregulated and 111 genes were downregulated in grafted cucumber. Above all, 68 genes were found to have significant differential expression levels in grafted cucumber compared with both self-rooted and failed grafted cucumber (**Table S3**), indicating that these genes could play a role in the pericarp wax biosynthesis of grafted cucumber. Based on the gene functions involved in wax biosynthesis and highest fold changes in grafted cucumber, 10 of the 68 DEGs were considered to be important candidate genes affecting pericarp wax formation (**Table 1**). These 10 DEGs had different functions associated with wax metabolism, including four DEGs involved in wax biosynthesis, three involved in wax transportation, and three AP2/ERF transcriptional factors that may be involved in the regulation of the gene expression of wax biosynthesis.

**Table 1:** The 10 differentially expressed genes involved in wax biosynthesis.

Gene category	Gene ID	FPKM			Gene Annotation
		SR^a^	G	FG	
Wax	Csa3G127750	3.5	57.6	3.0	CER1; contains fatty acid hydroxylase
	Csa6G079750	91.8	205.0	83.9	CER1 protein, putative, expressed
	Csa6G151810	6.1	28.7	9.0	CER4, fatty acyl-CoA reductase
	Csa6G302180	9.3	23.0	11.3	KCS1, 3-ketoacyl-CoA synthase
ABC/LTP	Csa3G446120	2.4	9.5	4.1	ABC transporter G family member
	Csa3G027200	14.5	42.1	33.8	14 kDa proline-rich protein DC2.15, putative
	Csa4G017140	154.5	214.3	104.6	PVR3-like protein
AP2/ERF	Csa6G496390	2.1	0.9	2.3	AP2-like ethylene-responsive transcription factor
	Csa3G878210	8.9	24.7	5.8	Ethylene-responsive transcription factor 1a
	Csa5G167110	6.7	20.8	15.5	Ethylene-responsive transcription factor

a SR, self-rooted; G, grafted; FG, failed grafted.

### Genomic Methylation Analysis of Pericarp Wax Synthesis After Grafting

A total of 4,247 methylated genes were detected in this study, 1,184 of which were specifically observed in grafted cucumber (as compared to self-rooted and failed grafted cucumber). Among these genes, the DNA methylated regions in the UTR, exon, intron, and promoter regions were distributed as 4, 48.8, 32.7, and 14.2% in the grafting-induced methylated genes, respectively. Twenty grafting-induced methylated genes were observed in the profile of differentially expressed genes in the grafted, but not in the un-grafted cucumber pericarp. These were identified as critical candidate genes affecting the changes in appearance of the scion pericarp in cucumber (**Table 2**). In contrast, we observed 2,521 methylated genes in the negative control but not in grafted treatment, these genes were assumed as the demethylated genes after graft, which included 43 DEGs based on transcriptome analysis (**Table S4**).

**Table 2:** List of 20 DEGs with DNA methylation in grafted cucumber.

Gene ID	FPKM			Gene Annotation
	SR^a^	G	FG	
Csa3G878210	24.7	6.7	15.5	Ethylene-responsive transcription factor 1a
Csa3G446120	9.5	2.4	4.1	ABC transporter G family member
Csa6G488880	15.6	10.1	7.4	Putative receptor-like protein kinase
Csa6G446400	10.8	7.2	4.8	Transcription factor, putative
Csa2G352420	38.4	7.9	26.3	Zinc finger CCCH domain-containing protein
Csa3G739050	23.8	11.4	17.2	Arabidopsis thaliana genomic DNA, chromosome 5, P1 clone:MOK16
Csa1G023030	35.8	29.8	16.4	Unknown protein
Csa3G817740	0.9	0.8	0.1	Probable exocyst complex component 6
Csa6G237600	109.1	52.3	104.4	Basic 7S globulin
Csa5G517100	5.5	5.3	2.6	Actin 4
Csa3G150000	117.1	50.2	113.1	Xyloglucan-specific endoglucanase inhibitor protein
Csa6G504490	6	5.9	2.1	ATP-dependent zinc metalloprotease FtsH 2
Csa7G048060	6.1	1.3	6.5	Plant-specific domain TIGR01615 family protein
Csa1G046270	3.5	5.1	7.3	Receptor protein kinase, putative
Csa5G199270	7.7	14.5	16.7	Aquaporin
Csa1G084320	116.9	297.7	226.9	Histone H4
Csa2G215520	2.1	5.8	1.6	Sulfate adenylyl transferase
Csa2G409480	2.3	5.6	6.6	Zinc finger family protein
Csa4G002500	0.4	1.2	1.1	Cellulose synthase-like protein
Csa2G196890	2	8.9	3.2	Sieve element occlusion protein 1

a Abbreviation was same as in **Table 1**.

The methylation sequencing results indicated that the intron region of *Csa3G878210* possesses a CCGG type methylation in grafted cucumber which was not detected in both negative control treatments. Furthermore, the expression of *Csa3G878210* was up-regulated in grafted cucumber compared with the self-rooted and failed grafted cucumber treatment. This gene is an AP2/ERF transcriptional factor and the homologous gene of the wax biosynthesis regulator gene *WIN1* (AP2/ERF gene) in *Arabidopsis*. Hence, this grafting-induced methylated gene (*Csa3G878210*) was named *CsWIN1*, which may play a critical role in regulation of wax biosynthesis in cucumber, especially in brightening the appearance of grafted pericarp, resulting in glossy cucumbers.

### Expression Validation of Wax Biosynthesis-Related Genes Affected by Grafting

In this study, the 10 obtained wax biosynthesis genes in transcriptome profiles showed considerable differences in gene expression levels between self-rooted and grafted cucumber pericarp. To confirm this results, qRT-PCR evaluation was performed in the self-rooted and grafted cucumber pericarp. There were eight genes (*CsCER1*, *CsCER1-1*, *CsCER4*, *CsKCS1*, *CsABC*, *Csa4G017140*, *Csa6G496390*, and *CsWIN1*) that were significantly (*p* < 0.01) upregulated in grafted cucumber, while one ERF gene (*Cs6G496390*) was downregulated. Thus, these results validated the transcriptome data (**Figure 3B**). However, one gene (*CsLTP*) showed a difference in expression level without significance. This validation result was consistent with the transcriptome profiles. Most of the wax biosynthesis-related genes were upregulated in the grafted cucumber pericarp (**Table 1**).

### Transcription Regulation Validation of *CsWIN1* in Pericarp Wax Biosynthesis

**Figure 4:**
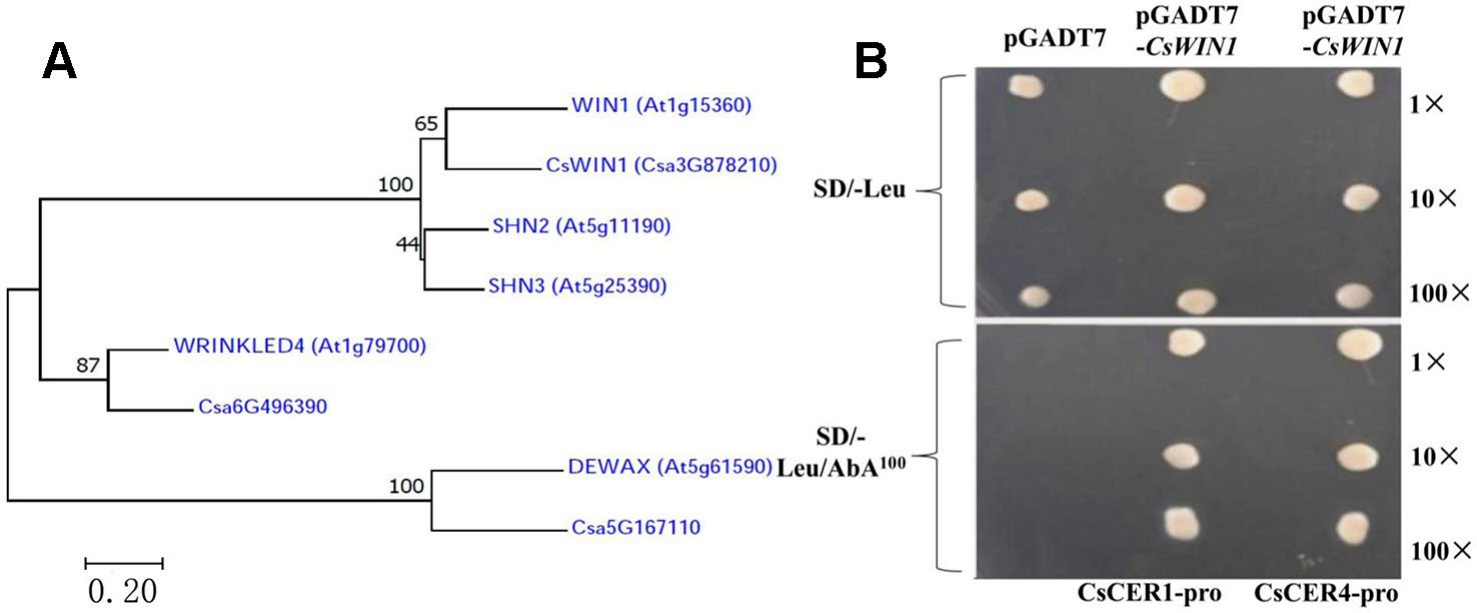
Phylogenetic analysis and Y1H transcriptional test of *CsWIN1* in the regulation of wax biosynthesis. **Figure 4A** displays the key wax regulator genes in *Arabidopsis* and their homologues in cucumber. **Figure 4B** shows the transcriptional binding test of *CsWIN1* with *CsCER1* and *CsCER4* promoters by Y1H.

In this study, five well-investigated AP2/ERF TFs in the regulation of wax biosynthesis in *Arabidopsis* were analyzed in the cucumber genome, and three homologue genes were obtained among the 68 DEGs identified in the transcriptome data, including the key *CsWIN*1 gene, which was specifically methylated by grafting in the cucumber pericarp, as well as two ERF genes. A phylogenetic tree aligned using the neighbor-joining (NJ) method through MEGA7 software were constructed and*CsWIN1* was confirmed to be closely related to the key wax biosynthesis regulator*WIN1*in *Arabidopsis* (**Figure 4A**) (Shi et al., 2011). In this study, the transcription regulation validation of *CsWIN1* was examined in the regulation of wax biosynthesis genes by a yeast one-hybrid system (Y1H). The promoter sequences of two targeted genes (*CsCER1*and *CsCER4*) including the element “GCCGGC” were amplified from genomic DNA and transformed into the pAbAi vector. The yeast cells that grew on the Y1H Gold-carrying PGADT7-*CsWIN1* vectors and the pBait-AbAi vectors could be grown in SD/-Leu/AbA 100 medium, and the positive clones increased gradually with the increase of the dilution concentration. This result indicated that pGADT7-*CsWIN1* harbors a transcriptional activation domain preferentially binding to the “GCCGGC” box of the *CsCER1* and *CsCER4* promoter (**Figure 4B**). The key grafting-induced regulator *CsWIN1* transcriptionally activates the expression of target wax biosynthesis genes, thus regulating the content and composition of pericarp wax in cucumber, resulting in the brightening and increased glossiness of the pericarp.

## Discussion

### The Unique Character of Cucumber Pericarp, as Well as Appearance After Grafting

In *Arabidopsis*, the wax appearance was crystal-like in the stem and leaves as well as in siliques (Haslam and Kunst, 2012). The chemical composition and content of cuticular wax is different among species and tissues (Kunst and Samuels, 2009).The cuticle wax responds to external environment stresses and is a physical and chemical barrier on the outer surfaces of terrestrial plants (Yeats and Rose, 2013; Ziv et al., 2018). The wax of cucumber fruit was observed as small balls or trichomes, quite different from that in *Arabidopsis* and other crops like wheat (Hen-Avivi et al., 2016). The small balls or trichomes in the glossy cucumber are integrated, while as those in waxy cucumbers appear broken and exist as fragmentary forms that are uniformly distributed on the surface of the cucumber fruit (**Figure 1B**). Previous studies also found that the chemical components of wax was different between *Arabidopsis* and cucumber, which phenols and alkenes were uniquely detected, while the ketones was unable to identified in cucumber (Wang et al., 2015b; Wang et al., 2015c).

In this study, we observed that after grafting with the rootstock of “Jingxinzhen 6”, the cucumber pericarp on the scion became glossy, as compared with the waxy appearance of the self-rooted cucumber (**Figure 1A**). The metabolite analysis indicates that there is a lower alkane content, but more wax esters were accumulated in the grafted cucumber.

Several studies have shown that the surface of cucumber fruit is more affected by silica, which is a natural powder covering on the surface of cucumber fruits, rather than the cuticular wax (Mitani et al., 2011). This finding was also investigated in the grafting system by comparing the effects of different rootstocks—*C. moschata* and *Cucurbita ficifolia*. The former pumpkin rootstock enhanced the glossy appearance of cucumber fruit, whereas the latter rootstock did not (Seki and Hotta, 1997). Another study showed that one amino acid mutation in the silicon influx transporter gene (AQP family genes) could lead to less silicon uptake in the soil, resulting in a glossy cucumber fruit when grafted onto *C. moschata* (Mitani et al., 2011). Here, we observed that four AQP family genes were all downregulated by grafting. These results are consistent with these previous studies (**Table S3**). Combined with the fact that the cuticular wax composition was significantly changed after grafting in this study, we hypothesized that there were two regulation pathways (wax biosynthesis and Si absorption) through which the rootstock affects the scion in grafting systems, though further research is required to validate this.

### Regulated Wax Biosynthesis Genes in the Pericarp After Grafting in Cucumber

Grafting is an important way to improve plant growth, stress tolerance, and fruit quality, and has been widely used in commercial horticultural crop production (Zahaf et al., 2012; Liu et al., 2016). The molecular mechanisms regulating plant growth *via* grafting have been investigated by several groups (Kyriacou et al., 2017), for example using high-throughput sequencing in watermelon (Liu et al., 2013), tomato (Estan et al., 2005), apple (An et al., 2018), and grapevine (Pagliarani et al., 2017). These studies showed that grafting affects differential expression of genes in grafted plants. miRNAs are also exchanged between the scion and rootstock in grafted watermelon, which may regulate the growth and development of the scion (Cookson and Ollat, 2013; Liu et al., 2013). A genomic DNA methylation analysis was performed in cucumber and melon scions by a grafting system using cucurbitaceous inter-grafting (Avramidou et al., 2015). Recently, some research identified many DEGs involved in various metabolic processes in grafted cucumber and verified by qRT-PCR (Miao et al., 2019a; Miao et al., 2019b). In this study, we observed that pericarp phenotype of cucumber grafted on pumpkin rootstock is distinct from these of the failed grafted cucumber and self-rooted cucumber. A previous study (Jun et al., 2016) also reported that the pericarp wax powder content in the self-grafted cucumber had no difference with self-rooted cucumber.

The present study identified 68 significantly DEGs in grafted cucumber and 10 of them were annotated in the wax biosynthesis pathway (**Table 1**). These 10 DEGs associated with wax biosynthesis were validated by qRT-PCR (**Figure 3B**). These DEGs may also function in VLCFAs biosynthesis and wax ester biosynthesis, as well as in the transportation of cuticular wax inside or outside of the cell in cucumber scion (McFarlane et al., 2010; Kim et al., 2012).

This study also examined the genomic methylation of cucumber scion grafted in pumpkin rootstock based on a genome-wide methylation sequencing method (Wang et al., 2015a), which was proved as a simple and scalable method. Although it may not detect all methylated genes in the genome because of limited sites by enzymes and the low coverage sequencing, it was a cost-effectively method in identifying candidate genes and had been used in many researches (Mastan et al., 2012; Rathore et al., 2014; Tang et al., 2014; Xia et al., 2014). Here, 20 key genes were methylated and the association analysis between the transcriptome and methylation identified one key wax biosynthesis regulator—*CsWIN1*, homologous to the ERF family members *WIN*, *WR4*, and *DEWAX* in *Arabidopsis*. The close phylogenetic relationship between *CsWIN1* and *WIN1* provides confirmation evidence that the ERF gene *CsWIN1* is an important regulator in wax biosynthesis. Moreover, this gene also affects expression of other genes (*CsCER1* and *CsCER4*) in the wax biosynthesis pathway, in addition to the downstream transporter gene *CsABC* in the cucumber scion.

### Potential Regulation Model of *CsWIN1* Affecting Pericarp Wax Biosynthesis

The key *CsWIN1* gene was observed in the transcriptome data and in the genomic DNA methylation profile. Furthermore, it is homologous to a key wax biosynthesis regulator in *DEWAX* in the model plant *Arabidopsis* (**Table S2**; **Figure 4A**). The expression of *CsWIN1* is positively correlated with four cuticular wax biosynthesis genes (*CsCER1*, *CsCER1-1*, *CsCER4*, and *CsKCS1*) that were upregulated after grafting on the pumpkin rootstock (**Figure 3**). In addition, the potential wax transporter genes (ABC/LTP transporter) were upregulated and co-expressed with the key regulator *CsWIN1* in the graft treatment (**Table S2**). Based on these results, we hypothesized that the key regulator *CsWIN1* affected by grafting could be a master switch that transcriptionally regulates the expression of the wax biosynthesis pathway genes in response to grafting, specifically activating *CsCER1*, *CsCER1-1*, *CsCER4*, and *CsKCS1* as well as transportation members of the ABC/LTP transporter family in the scion. TheY1H experiment validated that the key regulator could bind to the promoter region of *CsCER1* and Cs*CER4*, illustrating the feasibility of transcriptional regulation (**Figure 4B**). Moreover, the metabolite dataset showed that the content composition of cuticular wax the cucumber scion was significantly affected by grafting on pumpkin.

**Figure 5:**
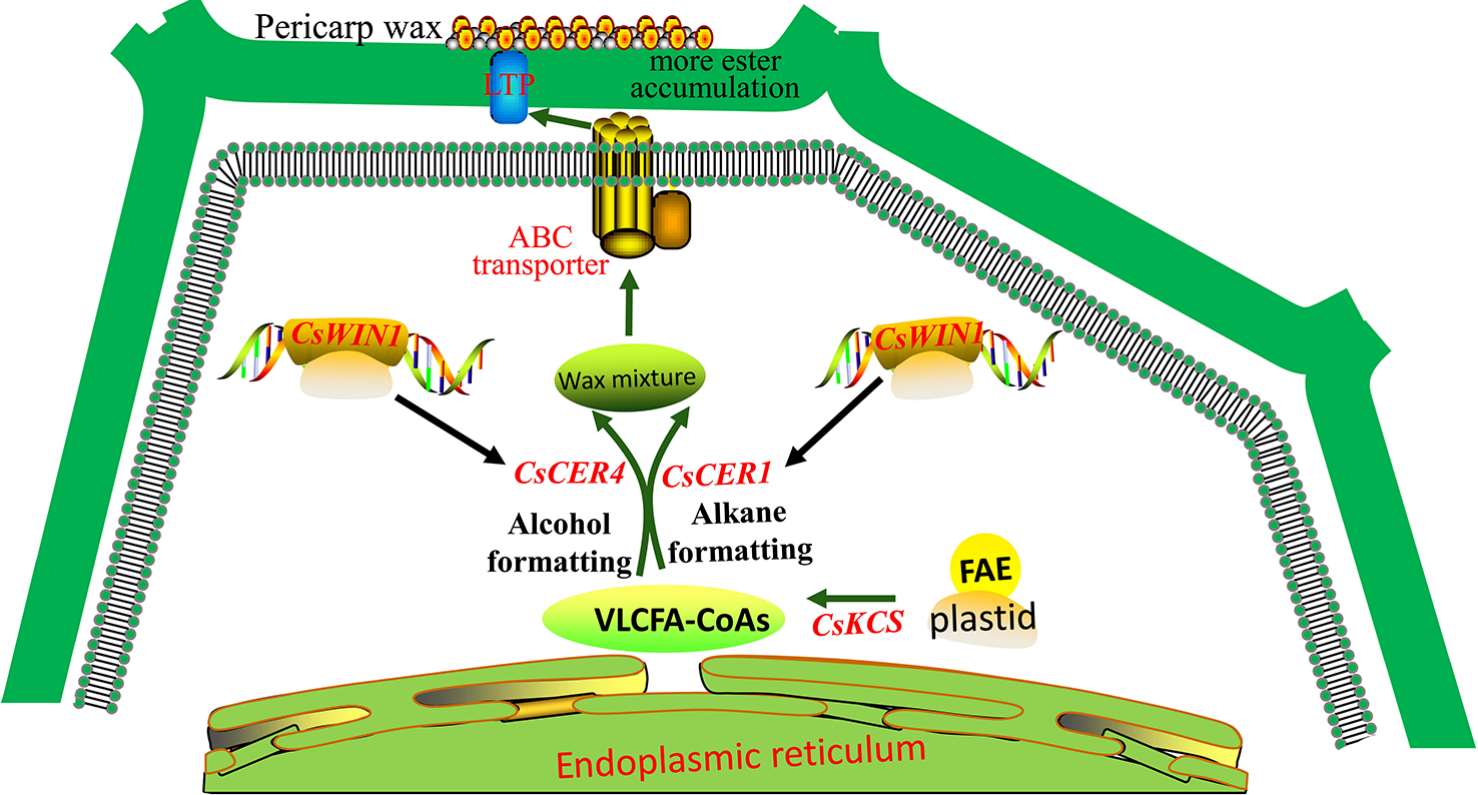
The potential regulation model of *CsWIN1* in wax biosynthesis. *CsWIN1* was methylated and upregulated by a grafting test. It then transcriptionally activated the expression of wax biosynthesis genes *CsCER1* and *CsCER4*, and may regulate the expression of transporter gene *CsABC*, resulted in the biosynthesis and transportation of more wax esters into the pericarp. This make the small trichomes not so fragile, so they do not break up in the grafted cucumber, as opposed to being easily broken in the self-rooted cucumber.

Following grafting, fewer alkanes and more wax esters were accumulated on the pericarp and the wax balls were not broken. Therefore, we propose a possible model of *CsWIN1* in the regulation of wax biosynthesis in grafted cucumber (**Figure 5**). *CsWIN1* was methylated and upregulated in grafted cucumber, and then transcriptionally activated the expression of wax biosynthesis genes *CsCER1* and *CsCER4*, and may regulate the expression of transporter gene *CsABC*, resulting in the biosynthesis and transportation of more wax esters into the pericarp. This process makes the small trichomes less prone to breaking in the grafted cucumber, as opposed to being easily broken in the self-rooted cucumber. This model shows a molecular regulation pathway of wax biosynthesis in cucumber and could provide a reference to other crops. However, the *in vitro* function of *CsWIN1* still needs to be validated by transformation experiments. The pumpkin’s genetic factors (genes, RNAs and proteins) that affect the cucumber pericarp are unknown and could be identified in future studies.

## Data Availability Statement

All datasets for this study are included in the article/**Supplementary Material**.

## Author Contributions

CW and YX designed this research. JZ and YY performed the grafting experiment, JY and JZ analyzed the bioinformatics data, and JL, JZ, and XZ performed the validation test. JZ, CW, and YX wrote the manuscript. All authors have read and approved the final manuscript.

## Funding

This research was financially supported by National Natural Science Foundation of China (No.31801887), China Postdoctoral Science Foundation (2018M631381), Beijing Postdoctoral Science Foundation (ZZ2019-49), Beijing Academy of Agricultural and Forestry Sciences (KJCX20170402/2018-ZZ-006/QNJJ201810), Beijing Nova Program (Z181100006218060), Beijing Municipal Department of Organization (2016000021223ZK22), and Beijing Youth Talent Promotion Project (2018).

## Conflict of Interest

The authors declare that the research was conducted in the absence of any commercial or financial relationships that could be construed as a potential conflict of interest.

## Supplementary Material

Figure S1 Heat map of all DEGs in self-rooted (SR), grafted (G), and failed grafted (FG) cucumber in three biological replications.

Table S1 All primer sequences used in this study.

Table S2 The basic analysis of RNA-seq reads.

Table S3 FPKM values of differentially expressed genes affected by grafting.

Table S4 List of 43 DEGs with DNA demethylation in grafted cucumber.
