# Supplementary figures and images for: Transcription Factor CsWIN1 Regulates Pericarp Wax Biosynthesis in Cucumber Grafted on Pumpkin
Source: Front Plant Sci. 2019 Nov 29;10:1564. doi: 10.3389/fpls.2019.01564 (PMC6895144; doi:10.3389/fpls.2019.01564)

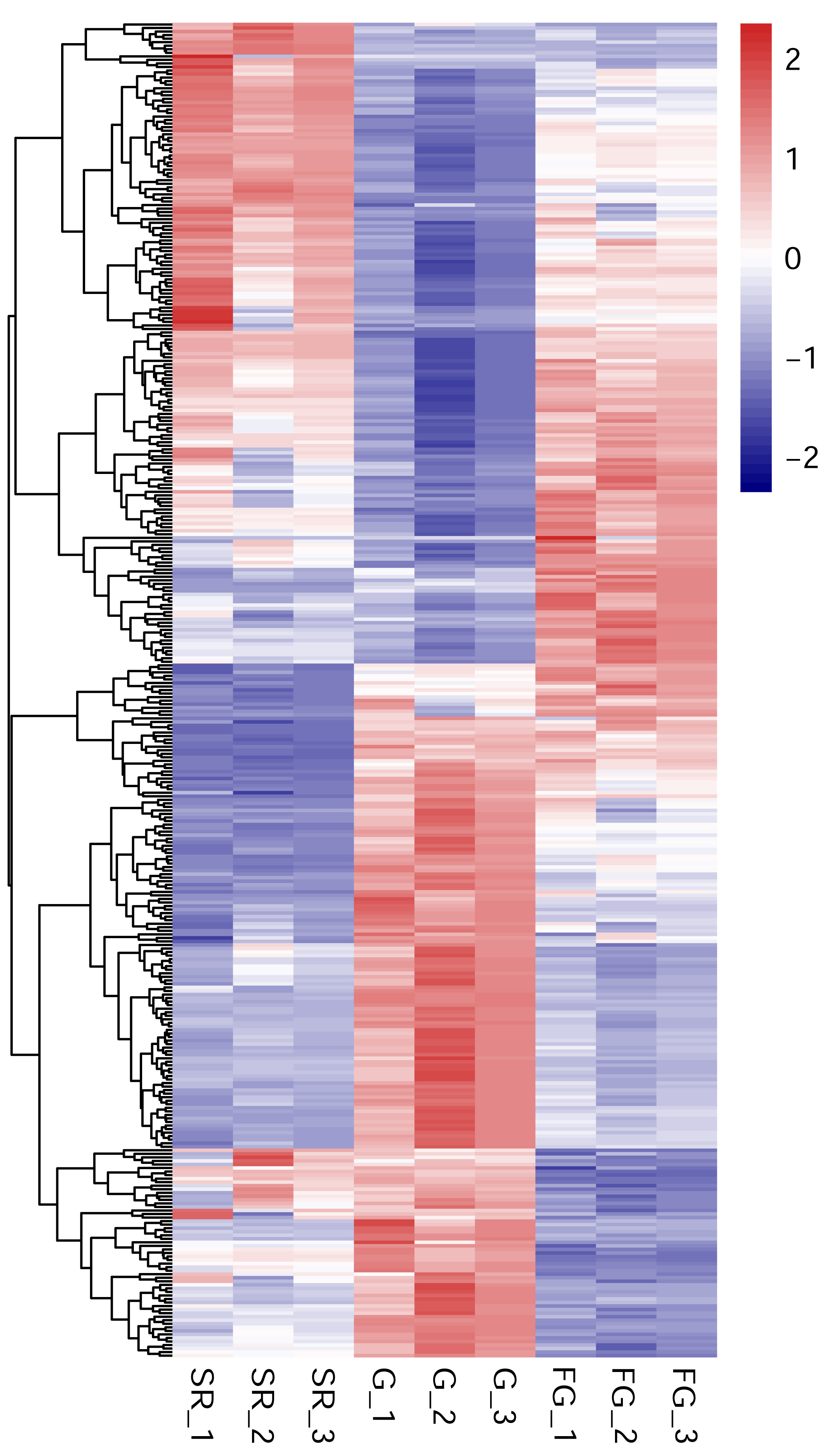

Supplement: Figure S1 — Heat map of all DEGs in self-rooted (SR), grafted (G), and failed grafted (FG) cucumber in three biological replications. [file Image_1.jpeg]
